# Patients’ experience with German primary care practices during Covid-19: an interview study

**DOI:** 10.3399/BJGPO.2023.0129

**Published:** 2024-03-20

**Authors:** Daniel Otto, Veronika van der Wardt

**Affiliations:** 1 Department of General Practice, University of Marburg, Marburg, Germany

**Keywords:** primary healthcare, general practitioners, patient experience, COVID-19, lockdown, health, qualitative research

## Abstract

**Background:**

Patient access to and communication with German primary care practices (PCPs) changed due to Covid-19. Patients had to comply with Covid-19 regulations, which included closed waiting rooms and appointment-based consultations. It is unclear how patients experienced these changes and how the pandemic impacted their primary care attendance.

**Aim:**

The aim of the study was to explore how patients, who frequently attended PCPs before the pandemic, perceived primary care during the initial phase of Covid-19 in Germany.

**Design & setting:**

Between January and June 2021, we completed 17 semi-structured interviews. Participants included primary care patients from two regions in Germany who frequently attended their physician before the start of the pandemic.

**Method:**

Data were analysed using content analysis.

**Results:**

Four interconnected themes emerged in the analysis: ‘fear of COVID-19 infection’, ‘practice organisation’, ‘information about COVID-19’, and ‘telemedicine’. Participants were unconcerned about being infected in their practice and mostly agreed with COVID-19 regulations, although waiting outside for their appointment was uncomfortable for some. Participants consulted their primary care physician in relation to different vaccines but felt they were sufficiently informed regarding general information about COVID-19. Views on telemedicine, which was mostly understood as contact via telephone or video call, differed widely, with some participants being very accepting and interested, while others dismissed telemedicine categorically.

**Conclusion:**

Participants regarded the new COVID-19 regulations as sensible. Telemedicine using telephone or video call consultations should be further explored under the assumption that this would be acceptable for some but not all patients.

## How this fits in

In German primary care practices (PCPs), strict Covid-19 regulations were implemented including wearing of masks, access only with appointment and waiting outside for the appointment instead of the waiting room. These regulations were mostly perceived as sensible and acceptable. Primary care physicians were consulted on vaccines but not for general information regarding COVID-19. Participants were divided on telemedicine using video or telephone calls; it was acceptable for some but unwelcome for others.

## Introduction

The COVID-19 pandemic rapidly changed how patients could access PCPs in Germany. Walk-ins were changed to telephone contacts or appointments, personal contacts with staff were reduced to a minimum, waiting rooms were closed, and patients were asked to wait outside, leading to long queues and waiting times.^1^ During lockdown fewer consultations were completed and consultations reasons changed, as did medication prescribed and services delivered.^2^ To date, the impact of the pandemic in primary care has been mostly investigated from the physician's perspective: studies examined the general experience of the pandemic across Europe,^1^ coping strategies for practices,^3^ the use of telemedicine and virtual consultations,^4^ the impact on training and professional identity development,^5^ financial effects,^6^ and patient contacts.^2^ Some studies also examined the patient's perspective, in particular their attitudes towards remote consultations: a Canadian cross-sectional study with 7532 participants found that primary care patients were comfortable with phone consultations (92%), video consultations (95%), and email or text messaging (91%).^7^ However, 31% of patients delayed care because of the pandemic, and patients who were aged ≥65 years, had lower financial status, had not been born in Canada, and/or had less education wanted to use these three digital consultation modalities significantly less beyond the pandemic, compared to other groups.^7^ Surveys conducted in the US showed similar high satisfaction rates with telemedicine,^8,9^ as did a large interview study with 66 participants from eight European countries during the first wave of COVID-19.^10^

A wider range of patient experiences was investigated in two interview studies: Homburg *et al* ^11^ showed that for Dutch patients, remote care was viewed as positive by some patients as it lowered the threshold for contacting the GP, but as negative by others as it increased the fear of missing a diagnosis and was experienced as less personal. The study also indicated mixed feelings about personal protective equipment (feeling of safety, less personal feeling), showed reduced accessibility to the GP but more time for scheduled visits and a quieter practice, and suggested that some postponed their check-ups for chronic conditions and were stressed about not being able to bring a relative to the consultation.^11^

Another interview study with patients, pharmacists, and a primary care physician^12^ investigated the experience of COVID-19 regulations in Irish PCPs. The findings indicated that many patients were reluctant to enter GP practices for fear of getting COVID-19 and other patients had difficulties getting appointments. Electronic prescriptions directly delivered to pharmacies were seen as useful and medication safety was not considered an issue by patients. Some of these aspects were reflected in a Scottish qualitative survey,^13^ which also showed frustrations about the difficulties getting an appointment and worries about catching COVID-19. Remote consultations by telephone or video were a positive experience by some, others lacked confidence in remote appointments.^13^

To date, it remains unclear how patients perceived COVID-19-related changes in Germany. Therefore, we explored how patients, who frequently attended PCPs before the pandemic, perceived and used primary care during the first year of COVID-19.

## Method

This study was a qualitative study based on semi-structured interviews with primary care patients.

### Participants

We recruited primary care patients who were aged ≥18 years and had at least nine contacts with the primary care physician (in person, by telephone, or video) within the 12 months before recruitment. We decided to include those who frequently attended the GP practice as it was assumed that these patients would be particularly affected by pandemic related changes. The definition of 'frequent attender' was based on a systematic review by Gill and Sharpe.^14^ Participants needed to be able to provide informed consent and have sufficient German language skills to understand the study information and complete the interview. COVID-19 infections were recorded but were not an eligibility criterion. Participants were recruited from PCPs who were willing to support the study in the German states of Hesse (North and Middle Hesse) and East Frisia. These states were initially chosen to include areas with different COVID-19 rates, but over the course of the study rates changed substantially, and a comparison of differently affected areas was no longer possible. We first contacted the practices via telephone to ask if they would be willing to support the recruitment of the study. If the leading GP agreed, we supplied them with information letters, consent forms, and the short questionnaire for the participants. Practices supporting the study displayed a poster outlining the study with contact details of the study team. Interested patients would first talk to their GP who would provide further information about the study and, optionally, the study information and consent form. Then, if patients were still interested, they would contact a member of the research team (DO) who introduced himself as the person who would also complete the interviews. We aimed for maximum variation sampling to include a diverse sample in terms of sex, age, and exposure to COVID-19.

### Data collection

Data were collected from January to June 2021. Interested primary care patients who called the study team and were eligible received the study information, the consent form (if they had not received it yet from the GP), and a short questionnaire by mail. The questionnaire asked about participants’ sex, age, living situation, if they or their relatives or friends had COVID-19 infections, and how often they visited their primary care physician within the last 12 months. Interested candidates could discuss the study and/or ask questions, either in person or by telephone. Both the signed consent form and the completed questionnaire were then sent back to the study team. All interviews were completed by the first author (DO) by telephone using a semi-structured interview guideline, which was developed by the research group based on the research aims and discussed in the department’s qualitative research method advisory group. The final version is available as Supplement 1. The interviewer (DO, male) was a medical student with interest in primary care who completed the project for his dissertation and had personal experience with COVID-19 during his placement in a GP practice. Guidance and training to complete the interviews was provided by VvdW, who is an experienced qualitative researcher. With the permission of participants, interviews were audio-recorded. Field notes were created during and after the interviews.

### Data analysis

Audio-recordings were transcribed verbatim using the transcription protocol by Dresing and Pehl.^15^ Transcriptions were analysed in combination with audio-recordings and field notes using a qualitative content analysis based on Kuckartz.^16^ Analysis started parallel to conducting further interviews. Translations of quotes were completed by VvdW, who is bilingual in German and English. A preliminary coding frame and potential themes were developed continuously, deductively guided by interview questions as well as inductively based on data. Once all interviews were completed, data were analysed by VvdW and DO independently. Subsequently the coding frames and themes were discussed within the research team and a qualitative study working group within the department of General Practice/Family Medicine. The data were revisited, themes were adapted, and the process was repeated. Preliminary results were presented to the qualitative study group of the Department of General/Family Medicine at the University of Marburg who provided feedback, which led to a final round of data-led refinement. The analysis was supported using the data management software MaxQDA (2022).^17^

## Results

In total, 17 primary care patients (7 women) participated in the study, with an age range between 20 and 88 years. Participants were recruited until a reasonably diverse sample, in terms of age, sex, and exposure to COVID-19, was achieved and coding did not generate new information that was considered relevant. Interviews took between 18 and 48 minutes. Participant pseudonyms and details are presented in Table 1.

**Table 1.** Study participants

	Sex	Age, years	Living situation	COVID-19 infection	Infections among family and friends	Primary care visits during the last year
ST1	Male	78	Living alone	No	Yes	5–9
STF2	Female	20	With partner	No	No	≥10
ST3	Female	45	Living alone	Yes	No	5–9
ST4	Male	59	With partner	No	Yes	≥10
ST5	Male	58	With partner	No	No	≥10
ST6	Female	88	Living alone	No	No	5–9
ST7	Female	65	With partner	No	No	5–9
ST8	Male	73	With partner	No	No	≥10
ST9	Male	67	with partner	No	No	≥10
ST10	Female	62	With partner	No	Yes	≥10
ST11	Male	67	With partner	No	No	≥10
ST12	Male	77	With partner	No	Yes	≥10
ST13	Male	83	With partner	No	No	5–9
ST14	Male	64	With partner	No	Yes	5–9
ST15	Female	61	With partner	No	No	≥10
ST16	Male	82	With partner	No	Yes	≥10
ST17	Female	76	With partner	No	Yes	≥10

Four interconnected main themes emerged in the analysis: ‘fear of COVID-19 infection’, ‘practice organisation’, ‘information about COVID-19’, and ‘telemedicine’.

### Fear of COVID-19 infection

Fear of a COVID-19 infection was discussed independently but also in the context of COVID-19 regulations in the primary care practice, which therefore became a sub-theme (‘COVID-19 regulations’). Another sub-theme of fear was ‘it’s not my personality’.

While fear of infection was discussed by all participants, their level of fear differed widely:

‘*No, I had never any concerns when I went to the doctor*.’ (ST4)

‘*No*, [I experienced fear] *more in supermarkets or public transport, not in the primary care practice.*’ (ST 8)

‘… *I had twisted my ankle. Usually, I would have gone to the doctor to have a look at it. But I didn’t. I didn’t want to go*.’ (ST6)

Reluctance to go to the physician was only partly due to fear; the sometimes difficult organisation of appointments also factored into the decision for some participants. Fear of COVID-19 in the PCP was more common in the beginning of the pandemic and had often passed at the point when the participant was interviewed:

‘*But then, no, then* [I was not afraid] *not anymore. Now, I would go anytime.*’ (ST3)

Familiarity with the PCP and trust were important. One participant regarded trust as a value for the PCP, which, in return, would create a responsibility to uphold the trust:

‘*And so there is trust. And in return, I’d say, the responsibility to maintain this trust. Therefore, I have no worries* [regarding the risk of infection].’ (ST8)

The busy environment of the practice did not increase the fear of contagion but was perceived as assurance that practice staff would be very careful as they were in danger of infections themselves.

#### Sub-theme: ‘It’s not my personality’

When participants considered their response to COVID-19 in the context of their primary care contacts, personality was used to explain their attitudes:

‘*I’m rather optimistic. I don’t have a pessimistic outlook on the world and always think it will be well. And I had no problems going into the primary care practice*.’ (ST1)

‘*I really had no fear; I’m not an overly fearful type*.’ (ST7)

#### Sub-theme: ‘COVID-19 regulations’

Strict implementation of and adherence to COVID-19 regulations, such as mask usage, use of disinfection dispensers, separate consultation hours for people with symptoms, and distancing rules mostly reassured patients and eased their worries about infections in the practice:

‘ … *yes, so, I am very happy with how the PCP deals with the situation. They* [PCP staff] *try to preferably eliminate the risk* [of infection].’ (ST4)

‘*So that* [the regulations] *showed me: OK, I am safe, and can regularly visit the doctor.*’ (ST15)

### Practice organisation

#### Sub-theme: ‘appointments’

For some people, getting appointments was easy; others had difficulties just getting into contact with the practice:

‘*It was difficult* [to get an appointment], *by phone, for example.*’ (ST9)

One person did not appreciate that she could not just walk in but had to make an appointment, which could not be changed with a quick chat to the receptionist. However, one person also mentioned that making an appointment was much easier during the pandemic. The participant suspected that fewer people tried to contact the practice.

#### Sub-theme: ‘waiting outside’

The ‘waiting outside’ rule, which many practices implemented to ensure distancing, was remarked on by many participants, some of whom did not approve:

‘… *and that was very uncomfortable for someone of my age, when you had to stand outside for long.*’ (ST6)

‘ … *when it rained … then it was not so good, that you had to stand outside.*’ (ST1)

On the other hand, there were participants who felt okay or safer waiting outside, and even some who preferred waiting outside already prior to the pandemic:

‘… *even before COVID-19, I did not like that, when you had to sit in the waiting room during flu season, you saw all the dripping eyes around you.*’ (S 11)

‘*I hate full waiting rooms. Also, in the PCP when we did not have a pandemic, I registered, and, when the weather was nice, said: “I’ll sit outside!”’* (ST9)

### Information about COVID-19

Most participants got their information about the pandemic from newspapers, television, and the internet. For many participants, there was too much information, which people experienced as overload and confusing:

‘*Covid-Extra, Covid-Special, Covid front, Covid sideways. It was all too much, ... because of the know-it-alls … also the virologists. That leads sometimes to uncertainty.*’ (ST9)

‘*I feel quite well informed. Partly over-informed.’* (ST11)

Information and assurance were also sought from online support groups. Primary care physicians were primarily consulted about questions relating to vaccines; participants partly assumed the physician would not know more than they did themselves:

*‘Which primary care physician would be able to answer these questions? He basically asks the same questions.*’ (ST 8)

### Telemedicine

Telemedicine was a topic raised by the interviewer, and therefore discussed in most interviews. Views on telemedicine focused on consultations via telephone or video calls, and ranged from welcoming to opposing:

‘*But I think this* [video consultations] *is actually a good idea.*’ (ST3)

‘… *it could become normality, I could very well imagine that*.’ (ST8)

‘*As long as it is not absolutely necessary, I’d rather not*.’ (ST6)

‘*No, that is not for me.*’ (ST11)

Several participants also wondered how telemedicine would work. However, while several people felt they would not like it for themselves, none of them were against it as a matter of principle for health care in primary care.

## Discussion

### Summary

The findings from interviews during the second and third wave of the pandemic reflect the initial worries and confusion, as well as their decline over the following year. In German PCPs, the COVID-19 regulations were accepted and perceived as a shield against the risk of an infection, providing safety for the patients and staff. Getting appointments was difficult for some but not for all. Even waiting outside was accepted, though for some it was uncomfortable, in particular in bad weather and if they had to stand for longer periods of time. Others, who already disliked waiting rooms before the pandemic, viewed it as a welcome escape. The participant’s own personality and general attitude was used to explain why they did not worry about COVID-19 infections. Given the quantity of information about COVID-19 from television, newspapers, and internet sources, participants rarely consulted their primary care physician even if they perceived the information as contradictory and confusing. Participants approached the physician only when they needed information regarding the range of vaccines. Telemedicine was understood as consultation via telephone or video call. Participants’ views differed widely, with some being very accepting and others dismissing it categorically.

### Strengths and limitations

The findings of this interview study are novel, as perceptions of COVID-19 in primary care have mostly been investigated from physicians’ but not patients’ perspectives. The study included a diverse sample in terms of sex, age, living situation, and experience of COVID-19 among family and friends, but only one participant had had a COVID-19 infection. Experiencing a COVID-19 infection might change the patient’s perception of the GP practice. For example, the fear of getting a new infection might decrease or increase worries about future infections and attitudes towards protection regulations. Furthermore, interviews were conducted during the start and height of the third wave of the pandemic in Germany; therefore, recall bias might have influenced the reporting of perceptions participants held during the earlier phases of the pandemic.

### Comparison with existing literature

Compared to the study done by Gleeson *et al*,^12^ our study findings showed similar anxiety in some patients about visiting the PCP. Some patients were reluctant to go to their GP during the initial phases of COVID-19, which is reflected in the results of an observational study that showed a reduction in GP visits of 49% during the first months of lockdown in Germany.^2^ Similar to the results of Brown *et al* ^13^ and Homburg *et al*,^11^ our findings indicated that getting an appointment was an issue for some of our participants. In addition, waiting outside was experienced as undesirable and uncomfortable for some, though not for everyone. It became evident in our study that, independent of the pandemic, some people would rather avoid sitting in a waiting room and were quite content waiting outside or going for a walk to pass the time until their appointment. Receiving live notifications for wait times, for example using pagers or texts, has been indicated to facilitate empathic care in a hospital setting.^18^ Similar systems using apps have shown to be useful and acceptable in a primary care setting.^19^ In line with general population data, our study reflected a declining fear of COVID-19 over time and the acceptance of regulations.^20^

Participants trusted their primary care physician, with trust having been identified as an important factor for compliance with COVID-19 preventive behaviours.^21^ This might explain the acceptance of COVID-19 regulations in the practices. The participants’ perceptions regarding information reflect evidence from a British survey assessing Covid-19 information overload,^4^ which indicated that the frequency of receiving information about the pandemic was associated with higher levels of fear and confusion. While clear COVID-19 information messages from PCPs might have helped patients who attended the practice regularly, it would require well-organised dissemination of knowledge to the GP practices as a German survey showed that GPs themselves felt that they lacked information.^22^ Telemedicine — which has received high levels of satisfaction by both physicians and patients,^9^ has demonstrated clinical empathy,^23^ and is considered to be feasible for primary care^24^ — was generally regarded more cautiously by the participants of this study. Some people supported it, some were unsure about it, and others did not consider it an alternative to face-to-face consultations. A recent literature review,^25^ reflected the mixed perception of telemedicine: some considered the remote consultations as convenient and a sensible measure to avoid COVID-19 infections, others had concerns about privacy, felt they lacked technical skills, worried about additional costs if the patient had to subsequently have a face-to-face appointment, and expressed barriers due to communication issues, loss of physical assessments, loss of non-verbal communication and shorter, or missed, consultations. Considering the range of issues and the reluctance of some German patients to use telemedicine, remote consultations should be introduced gradually and with appropriate support. Patients could be provided with information on how to participate in a remote consultation and, if needed, shown how to use a mobile phone, tablet, or computer for that purpose in the practice.

### Implications for research and practice

While fear and worries declined during the course of the pandemic and acceptance of COVID-19 regulations in the PCP was high, information overload was a large contributor to worries and confusion. Clear and distinct health information from PCPs might have helped to ease the feeling of information overload and confusion. Waiting rooms, which have been standard in PCPs before the pandemic, should be re-examined, as some people would rather avoid them. Using digital technology to provide live notifications (such as texts or pagers) when appointments are starting would support those patients who do not want to use the waiting room. Remote consultations should be introduced gradually and with support from the PCP, in particular for those who are not confident using digital technology. To increase preparedness for pandemic outbreaks, research should explore how patients who are not confident in using digital technology could be supported to participate in remote consultations, and who could provide this support.

## Data

Data are not available as this has not been included in the consent form.
